# The Intersection of Migraine and Epistaxis: Clinical Observations and Analysis

**DOI:** 10.7759/cureus.65584

**Published:** 2024-07-28

**Authors:** Shalesh Rohatgi, Salil Gundewar, Satish Nirhale, Prajwal Rao, Pravin Naphade, Arun B Oommen, Prashant Dubey, Advait A Gitay, Pranit Khandait

**Affiliations:** 1 Department of Neurology, Dr. D Y Patil Medical College, Hospital and Research Centre, Pune, IND; 2 Department of General Medicine, Dr. D Y Patil Medical College, Hospital and Research Centre, Pune, IND

**Keywords:** international headache society, headache, trigeminovascular system, epistaxis, migraine

## Abstract

Migraine prevalence has risen over the last few decades, which may be attributed to lifestyle changes. Epistaxis is unusual in migraine. Here we present a case series of four patients, who are presented with headaches associated with epistaxis. A detailed history revealed cardinal symptoms of migraine according to the International Headache Society, including hemicranial throbbing headache of moderate to severe intensity lasting for a duration of four to 72 hours, along with associated features of nausea, vomiting, photophobia, and phonophobia. Investigations, including ENT (ear, nose, and throat) examination, nasal endoscopy, gastroscopy, bronchoscopy, hematological, and coagulation parameters, were negative. All patients were started on prophylactic treatment for migraine, and they responded well. Epistaxis occurs at the peak of headache following which symptoms tend to resolve. The pathophysiology behind this is stimulation of the trigeminovascular system leading to dilatation of external and internal carotid arteries.

## Introduction

Migraine is one of the most frequently encountered types of headache, with a global incidence of 87.6 million cases [1]. The prevalence of migraine-associated epistaxis is 11.8%, which occurs due to stimulation of the trigeminovascular system, causing vasodilation of the internal and external carotid arteries and their branches [2]. Typical migraine presentations include unilateral throbbing headaches with associated symptoms such as nausea, vomiting, photophobia, and phonophobia [3,4]. Migraine attacks typically progress through four distinct phases: prodrome, aura, headache, and postdrome. Each phase is characterized by specific symptoms and changes in neurological function, with the headache phase often being the most debilitating, marked by intense pain and associated symptoms such as nausea and photophobia [5]. Diagnostic methods involve clinical evaluation and exclusion of secondary causes through imaging and other relevant investigations [6,7]. Differential diagnoses include other primary headache disorders, sinusitis, vascular anomalies, and paroxysmal hemicranial headache [8]. Treatment strategies encompass both abortive and prophylactic medications [9]. Emerging therapies focus on calcitonin gene-related peptide (CGRP) antagonists and neuromodulation techniques [10,11]. We present four cases of migraine associated with epistaxis. All other causes of epistaxis were ruled out through relevant investigations. All patients had classical migraine without aura and responded well to abortive and prophylactic therapy.

## Case presentation

We present four patients, aged 17 to 34 years, who experienced migraines associated with epistaxis. These cases include both males and females, all with a history of hemicranial throbbing headaches. Each patient underwent a comprehensive workup to exclude other causes of epistaxis, including coagulation profiles, ENT (ear, nose, and throat) evaluations, and imaging studies. All patients responded well to migraine prophylactic treatment, including drugs such as beta-blockers (propranolol) and calcium channel blockers (flunarizine). They did not experience any further episodes of migraine or epistaxis during the six-month follow-up period.

Table 1 summarizes the frequency, severity, and presence of auras among the four patients. It is noted that none of the patients experienced an aura. Two patients had daily headaches, while the other two experienced headaches three to five times per week. Headache severity varied with two patients experiencing severe headaches and two experiencing moderate headaches.

**Table 1:** Summary of headache frequency, presence of aura, headache severity, and migraine phase among the four patients

Patient	Headache frequency (per week)	Aura	Headache severity	Phase of migraine
1	3-4	No	Moderate	Headache
2	4-5	No	Severe	Headache
3	Daily	No	Severe	Headache
4	Daily	No	Moderate	Headache

Case 1

An 18-year-old female with no known comorbidities was admitted with complaints of a headache along with episodes of bloody vomiting and blood in sputum for the last three months. Blood in the vomitus was bright red. She underwent multiple investigations, including a coagulation profile, bleeding time, clotting time, ENT evaluation including nasal endoscopy, computed tomography (CT) scan of the paranasal sinuses, gastroscopy, and bronchoscopy, all of which were normal. A detailed history was suggestive of episodic left hemicranial throbbing headache lasting for three to four hours, associated with photophobia and phonophobia, with immediate relief in headaches post-bleeding from the nose or vomitus of swallowed blood. Fundus examination was normal. MRI brain and MR venography were normal. Antineutrophil cytoplasmic antibody (ANCA) was negative. The patient was started on migraine prophylaxis, following which she had significant relief of symptoms.

Case 2

A 34-year-old female with no known comorbidities presented with complaints of a headache for the last two months, associated with epistaxis. The headache was a right hemicranial throbbing type, lasting for two to three hours, associated with nausea and vomiting. It used to get relieved after an episode of epistaxis. The patient used to have four to five episodes of a headache every week. Neurological examination was normal. MRI brain venography was normal. Detailed evaluation, including a coagulation profile, bleeding time, clotting time, ENT examination including nasal endoscopy, and CT of paranasal sinuses, were normal. The patient was started on migraine prophylaxis, following which she had complete relief of symptoms.

Case 3

A 17-year-old male with no known comorbidities was admitted with complaints of a headache for the last six months, associated with episodes of epistaxis. History was suggestive of episodic, right occipital headache, severe intensity, throbbing type, and lasting for three to four hours. He used to have seven to 10 episodes per month initially. Later the frequency increased to daily headaches for 15 days. Bleeding episodes used to occur at the peak of the headache followed by the resolution of symptoms. Examination, including fundus examination, ENT examination, and neurological examination, was within normal limits. MRI brain venography was normal. Detailed evaluation, including a coagulation profile, bleeding time, clotting time, nasal endoscopy, and CT paranasal sinuses, were within normal limits. After starting the patient on migraine prophylaxis, he had complete relief of symptoms.

Case 4

A 31-year-old male with no known comorbidities presented with complaints of a headache for the last six months. The headache was episodic, right hemicranial, throbbing type, each episode lasting for three to four hours, and associated with photophobia. He used to have around two to three episodes daily. The patient used to be asymptomatic in between the episodes. Epistaxis used to occur at the peak of the headache. The patient used to have relief from headaches after this. ENT examination, including nasal endoscopy, CT paranasal sinuses, esophagogastroduodenoscopy, coagulation profile, bleeding time, and clotting time, were normal. Neurological and fundus examinations were normal. MRI brain venography was normal. After starting the patient on migraine prophylaxis, he had complete relief of symptoms.

## Discussion

Migraine, derived from the Greek word "hemikrania," affects 12% of the global population, with a higher prevalence in females [1,2]. Migraine without aura is the most common type, representing 75% of cases [3,4]. The trigeminovascular theory, proposed 40 years ago, suggests that stimulation of the trigeminovascular system releases vasoactive peptides such as substance P, CGRP, and pituitary adenylate cyclase-activating polypeptide (PACAP) causing vasodilation of the carotid arteries [6,7].

Epistaxis at the headache peak suggests symptom resolution through secretion, which could also include vomiting, diuresis, lacrimation, and sweating [8,9]. A detailed history and evaluation are crucial to rule out organic causes and reassure patients of the benign nature of their symptoms [10,11]. Patients with migraine-associated epistaxis tend to experience relief of symptoms with appropriate headache management [12,13].

Migraine pathophysiology involves the activation and sensitization of the trigeminovascular system, where CGRP plays a crucial role. CGRP is released from trigeminal nerve endings, leading to vasodilation and pain experienced during migraine attacks [14]. Elevated levels of CGRP during attacks, along with its ability to trigger migraine-like symptoms, further emphasize its involvement in migraine pathophysiology [15]. The release of CGRP initiates a cascade of events, including increased nitric oxide synthesis and trigeminal nerve sensitization, perpetuating peripheral and central sensitization processes [16]. Additionally, ion channels have been identified as important participants in migraine pathogenesis, affecting neuronal excitability and pain sensation within the trigeminovascular system [17]. Understanding these mechanisms is crucial for developing effective treatments targeting CGRP and ion channels to alleviate migraine symptoms and improve patient outcomes.

The relationship between migraine and epistaxis can be explained by the trigeminovascular system's role in migraine pathophysiology. Activation of this system leads to the release of neuropeptides that cause vasodilation and increased vascular permeability, potentially leading to epistaxis [18,19]. The vascular branches supplying nostrils are internal maxillary arteries arising from the external carotid artery and ethmoidal arteries arising from the internal carotid artery. The maxillary nerve and ethmoidal nerve are branches of the trigeminal nerve which further forms a part of the trigeminovascular system. These nerves supply the maxillary and ethmoidal arteries causing their vasodilatation and promoting epistaxis at the peak of migraine headache [20]. In a study by Das et al., the results suggest a higher incidence of epistaxis in migraine patients emphasizing the importance of recognizing this co-occurrence in clinical practice for appropriate management and treatment strategies [21].

Prophylactic treatments for migraine, such as beta-blockers and calcium channel blockers, have shown efficacy in reducing headache frequency and associated symptoms [22,23]. Emerging therapies targeting CGRP and PACAP pathways show promise in treating migraine with associated symptoms [24,25]. Further research is needed to explore the mechanisms underlying migraine-associated epistaxis and to develop targeted treatments that address both headache and epistaxis in these patients [26,27].

## Conclusions

In this case series, we explored the unusual association of epistaxis with migraine. Due to the activation of the trigeminovascular system, migraine may rarely be associated with epistaxis. A thorough evaluation should be performed to rule out other critical causes of epistaxis. With effective treatment and prophylaxis of migraine headaches, epistaxis episodes tend to resolve completely. Thus patients and relatives should be counseled regarding the benign nature of the symptom as it may trigger anxiety and panic among them. Newer therapies like CGRP antagonists could be tried for effective prophylaxis of migraine in these cases. 
